# Identification and validation of novel lung adenocarcinoma subtypes and construction of prognostic models: based on cuprotosis-related genes

**DOI:** 10.1186/s12890-023-02350-6

**Published:** 2023-02-11

**Authors:** Guangyao Wang, Anqiao Wang, Li Wang, Guanglan Xu, Xiaohua Hong, Fang Fang

**Affiliations:** 1 The First Affiliated Hospital of Guangxi University of Chinese Medicine, Nanning, 530000 China; 2 Longgang District People’s Hospital of Shenzhen, Shenzhen, 518038 China; 3 Guangxi University of Chinese Medicine, NanNing, 530000 China

**Keywords:** Cuprotosis, Lung adenocarcinoma, Prognosis, Risk profile, Immune infiltration

## Abstract

Cuprotosis is a novel and unique form of cell death that is of great value in a variety of cancers. However, the prognostic role of cuprotosis-related genes (CRGs) in lung cancer remains undetermined. We compared the expression profile of CRGs in lung adenocarcinoma (LUAD) patients, revealing the genetic alterations and inter-gene correlations of CRGs. Based on 13 CRGs, LUAD patients could be well differentiated into two molecular subgroups, and the differentially expressed genes (DEGs) in these molecular subtypes were identified. Furthermore, 10 cuprotosis pattern-related DEGs with a significant prognostic value were obtained for constructing a prognostic model. Through validation in an external validation set, the prognostic model based on the CRGs-risk score showed the robust and effective predictive ability and served as an independent prognostic indicator for LUAD patients. Therefore, combining the CRGs-risk score with multiple factors such as clinicopathological characteristics, a quantitative nomogram was developed to predict the survival and prognosis of LUAD patients, improving the clinical application value of the CRGs-risk score. In the low CRGs-risk score group, the related immune cell infiltration was increased and the immune function was activated in LUAD patients. This study may add to the knowledge of CRGs in LUAD, partly contribute to evaluating the prognosis of LUAD patients, and provide direction for the development of targeted therapy and immunotherapy.

## Introduction

Lung cancer, the major cause of cancer-related death in both males and females, shows a rising incidence in most countries across the world. Despite continuous updating and improvement of therapeutic methods, the prognosis of lung cancer patients is still not optimistic due to the heterogeneity and complexity of the disease [1, 2]. Currently, the most common predisposing factors for this cancer consist of smoking [3], environmental factors (air pollution, occupational exposure) [4], hereditary factors (lung cancer susceptibility genes) [5], ethnic variations [6], etc. Most lung cancers are diagnosed in the advanced stage cancer. A study noted that the 5-year survival rate of patients diagnosed with lung cancer worldwide has been only approximately 15% in the past 10 years [7]. The cancer-related burden of lung cancers is projected to double by 2050 [8]. Therefore, early screening and comprehensive risk assessment are of great significance for lung cancer patients. However, the significant heterogeneity of lung cancer seriously affects the accuracy of existing clinical assessment tools [9]. Genomic testing has become popular and is conducive to identifying gene mutations within heterogeneous lung tumours, which is helpful for the determination of clinical treatment plans and evaluation of patient prognosis [10]. To improve the prognostic judgement accuracy for lung cancer patients, it is necessary to construct novel genetic prognostic models applicable to clinical demands.

It has been revealed that multiple forms of cell death represent an important aspect in exploring antitumour therapies [11]. For instance, ferroptosis, pyroptosis, and necroptosis offer new insights into the treatment of tumours. Cuproptosis, a novel and unique form of cell death, was recently discovered to be different from other types of cell death, such as necroptosis and pyroptosis. The onset of cuproptosis depends on the cellular accumulation of copper and is regulated by mitochondrial respiration. After direct binding to fatty acylated proteins, aggregation of fatty acylated proteins leads to loss of iron-sulfur protein, which eventually causes cell death owing to proteotoxic stress. During this process, FDX1, an upstream mediator of protein fatty acylation, acts as a key modulator of cuproptosis [12]. Cell death induced by the imbalance of copper homeostasis in vivo is of great value in a variety of cancers, including lung cancer. At present, several copper-related agents, such as quinoline and tetrathiomolybdate, have been applied in the treatment of cancers and have shown promising anticancer activity [13, 14]. With in-depth exploration, Kadu P et al. revealed that copper-lowering agents can enhance the antitumour activity of chemotherapeutic drugs [15]. Fortunately, many recent studies have focused on the relationship between cuproptosis, tumour typing and the tumour microenvironment. Moreover, Liu et al. [16] found that lung adenocarcinoma (LUAD) types with high tumour mutation burden (TMB) have a good prognosis and good immune function. However, the above study only used TCGA data for analysis and did not use GEO data for further verification. Moreover, there is still no risk scoring system for predicting the prognosis of LUAD patients based on cuproptosis-related genes (CRGs).

In this study, we comprehensively evaluated CRGs, determined the differential expression profiles of CRGs in LUAD tissues and normal tissues, and analysed the mutation status and intergene correlations of CRGs in LUAD. Additionally, LUAD patients were assigned into two molecular subgroups depending upon the expression patterns of CRGs, and then differentially expressed genes (DEGs) between the two molecular subgroups were identified. After screening the CRGs with significant prognostic value, we constructed a risk scoring system (the CRG risk score) for LUAD patients, which can accurately predict the prognosis of LUAD patients. Moreover, we established a prognostic nomogram by integrating the risk score with clinicopathological characteristics. Ultimately, the immune status of LUAD patients in the high and low risk score groups was described.

## Materials and methods

### Data sources and preprocessing

**Fig. 1:**
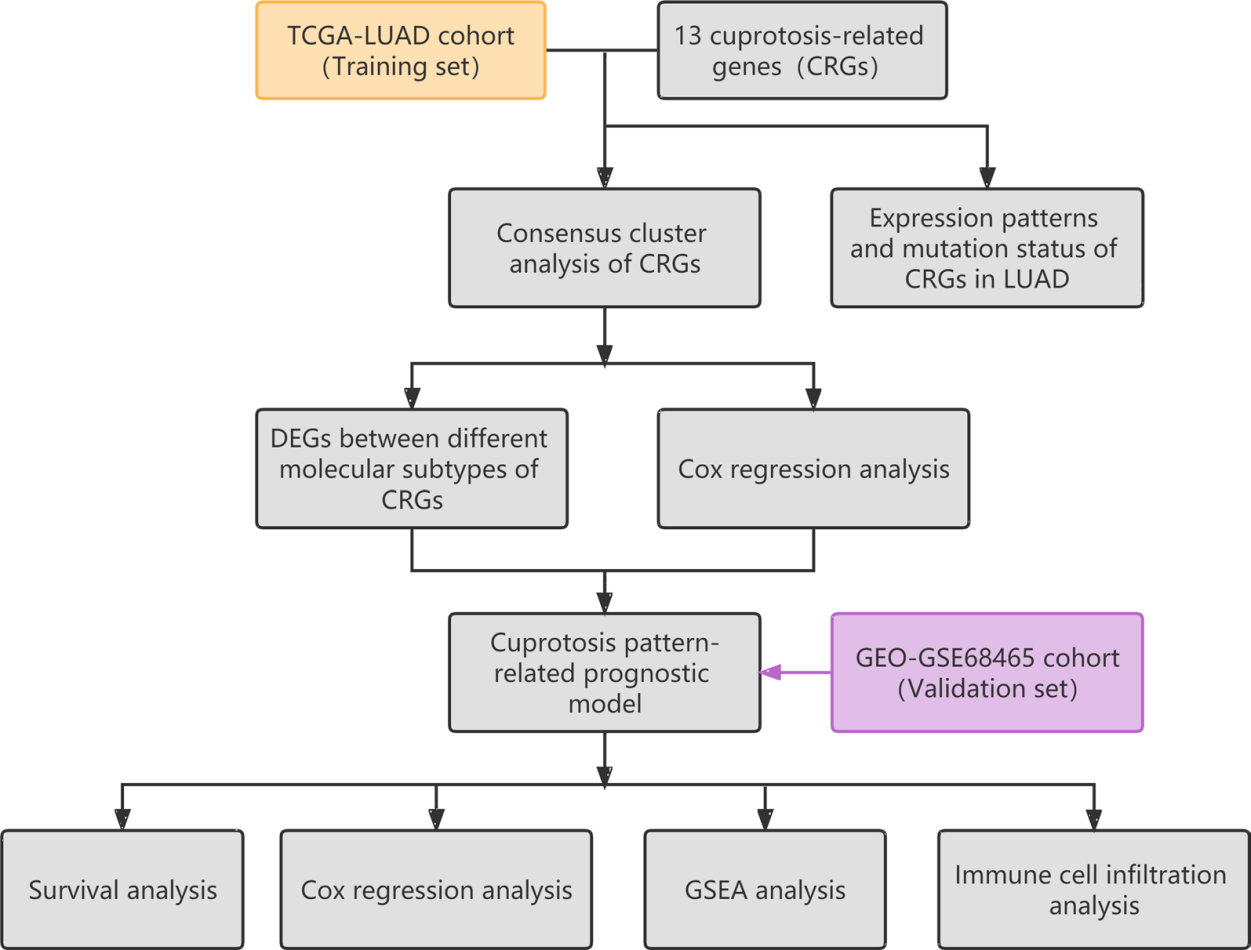
Schematic outlining the workflow of this study

The workflow of this study is outlined in Fig. 1. The gene expression matrix of the LUAD cohort and relevant clinicopathological and prognostic data were downloaded from TCGA and GEO websites (TCGA, https://portal.gdc.cancer.gov/; GEO, https://www.ncbi.nlm.nih.gov/geo/). TCGA-LUAD cohort (training set) and the GEO-GSE68465 cohort (validation set) were utilized for all subsequent analyses. The patient information with missing values or a survival time of 0 was deleted from the corresponding clinicopathological prognostic data of TCGA-LUAD cohort, followed by the normalization of gene expression data from the GEO-GSE68465 cohort. The clinical characteristics of LUAD patients are mentioned in detail in Table 1. This study was exempted from ethical review because we used publicly available data from TCGA and the GEO website.

**Table 1:** Clinical characteristics of the patients with LUAD used in this study

Characteristics	TCGA (training set, N = 509)	GSE68465 (validation set, N = 443)	Total (N = 952)	*p* value	FDR
Time					
Mean ± SD	909.60 ± 892.04	1578.13 ± 1087.35	1220.31 ± 1041.95		
Median[min–max]	658.00 [4.00,7248.00]	1410.00 [0.90,6120.00]	900.00 [0.90,7248.00]		
State				1.10*10^–7^	4.50*10^–7^
Alive	326 (34.24%)	207 (21.74%)	533 (55.99%)		
Dead	183 (19.22%)	236 (24.79%)	419 (44.01%)		
Age					
Mean ± SD	65.29 ± 10.03	64.42 ± 10.10	64.88 ± 10.07		
Median[min–max]	66.00 [33.00,88.00]	65.00 [33.00,87.00]	65.50 [33.00,88.00]		
Sex				0.22	0.33
Female	274 (28.78%)	220 (23.11%)	494 (51.89%)		
Male	235 (24.68%)	223 (23.42%)	458 (48.11%)		
T				0.17	0.33
T1	171 (17.96%)	150 (15.76%)	321 (33.72%)		
T2	272 (28.57%)	251 (26.37%)	523 (54.94%)		
T3	45 (4.73%)	28 (2.94%)	73 (7.67%)		
T4	18 (1.89%)	12 (1.26%)	30 (3.15%)		
TX	3 (0.32%)	0 (0.0e + 0%)	3 (0.32%)		
NA	0 (0.0e + 0%)	2 (0.21%)	2 (0.21%)		
N				0.06	0.18
N0	329 (34.56%)	299 (31.41%)	628 (65.97%)		
N1	97 (10.19%)	88 (9.24%)	185 (19.43%)		
N2	70 (7.35%)	53 (5.57%)	123 (12.92%)		
N3	2 (0.21%)	0 (0.0e + 0%)	2 (0.21%)		
NA	0 (0.0e + 0%)	2 (0.21%)	2 (0.21%)		
NX	10 (1.05%)	1 (0.11%)	11 (1.16%)		
NA	1 (0.11%)	0 (0.0e + 0%)	1 (0.11%)		
M				4.20*10^–200^	2.50*10^–199^
M0	341 (35.82%)	0 (0.0e + 0%)	341 (35.82%)		
M1	17 (1.79%)	0 (0.0e + 0%)	17 (1.79%)		
M1a	2 (0.21%)	0 (0.0e + 0%)	2 (0.21%)		
M1b	5 (0.53%)	0 (0.0e + 0%)	5 (0.53%)		
MX	140 (14.71%)	0 (0.0e + 0%)	140 (14.71%)		
NA	4 (0.42%)	443 (46.53%)	447 (46.95%)		
Stage				6.30*10^–198^	3.10*10^–197^
Stage I	273 (28.68%)	0 (0.0e + 0%)	273 (28.68%)		
Stage II	122 (12.82%)	0 (0.0e + 0%)	122 (12.82%)		
Stage III	81 (8.51%)	0 (0.0e + 0%)	81 (8.51%)		
Stage IV	25 (2.63%)	0 (0.0e + 0%)	25 (2.63%)		
NA	8 (0.84%)	443 (46.53%)	451 (47.37%)		

### Expression patterns and mutation status of CRGs in LUAD

Tsvetkov P et al. [12] identified 13 CRGs that we used for this study, and the corresponding specific details are presented in Table 2. We conducted a mutation analysis to understand the regulation of genetic variation of CRGs in LUAD. Meanwhile, we also compared the differential expression of CRGs in normal lung and LUAD tissues to evaluate the effect of change in their expression on mRNA levels. A heat map of TCGA-LUAD cohort was visualized by the "pheatmap" package of the R software to analyze the expression and mutation status of 13 CRGs in LUAD. The protein–protein interaction and gene co-expression networks of 13 CRGs were visualized using the STRING online database and the "igraph, reshape2" package of the R software. In addition, the mutation status of CRGs in LUAD was analyzed utilizing the "maftools" package of the R software.

**Table 2:** Summary of 13 recognized cuprotosis-related genes

Gene	Type
FDX1	Cuprotosis
LIPT1	Cuprotosis
LIAS	Cuprotosis
DLD	Cuprotosis
DBT	Cuprotosis
GCSH	Cuprotosis
DLST	Cuprotosis
DLAT	Cuprotosis
PDHA1	Cuprotosis
PDHB	Cuprotosis
SLC31A1	Cuprotosis
ATP7A	Cuprotosis
ATP7B	Cuprotosis

### Consensus cluster analysis of CRGs and their relations to clinical status and prognosis

The consensus clustering of CRGs was carried out employing the "limma, survival, ConsensusClusterPlus" package of R software. LUAD patients were stratified into different molecular subgroups based on the level of expression of the 13 CRGs. In this study, we compared clinicopathological features (age, gender, TNM/Stage classification) and prognosis of TCGA-LUAD patients in molecular subgroups to analyze their correlations to determine the clinical value of the two molecular subtypes obtained by the CRG-based consensus clustering analysis. Meanwhile, the "survival, survminer" package of the R software plotted the survival curves of the two molecular subtypes. Moreover, the survival differences between the two molecular subtypes were evaluated.

### DEGs between different molecular subtypes of LUAD

Identifying differentially expressed genes (DEGs) between the different molecular subtypes was critical for subsequent studies. The DEGs between different molecular subtypes of CRGs were selected using the "limma, ggplot2" package of the R software with the selection criteria as follows: (1) logFC = 0.585; (2) FDR = 0.05; (3) *p-value* < 0.05. We also performed gene ontology and pathway enrichment analyses on cuprotosis-related DEGs using the "clusterProfiler, org.Hs.eg.db, enrichplot, ggplot2" R packages to further investigate the potential biological functions and signaling pathways.

### Construction and validation of a cuprotosis pattern-related prognostic model

The risk score of each LUAD patient in a cuprotosis-related prognostic model was calculated to quantify the cuprotosis patterns. Univariate Cox regression analysis (“survival” R package) screened the cuprotosis pattern-related DEGs with prognostic values, with the significance level being < 0.05. The selected genes were enrolled for further LASSO regression analysis ("glmnet, survival" R package) to eliminate the risk of overfitting maximally. The remaining final prognostic genes were chosen for constructing the model whose risk score was calculated using the following formula: CRG-risk score = $\sum {_{i - 1}^{n} {\text{coefi}} \times {\text{gene}}\_{\text{expressioni}}}$, wherein coef refers to the regression coefficient, and gene_expression refers to the expression of each prognostic gene. The LUAD patients in the training set were divided into high- and low-risk subgroups according to the median value of the CRG-risk score. Then, Kaplan–Meier survival analysis was performed on the two risk subgroups using the "survival, survminer" package of the R software. Afterward, principal component analysis (PCA) on the two risk subgroups was carried out with the "ggplot2" R package, and the risk distribution graphs and receiver operating characteristic (ROC) curves of the two risk groups were generated using the "pheatmap, survival, survminer, timeROC" R package. Moreover, the independent prognostic value of the CRG-risk score was evaluated by performing uni- and multivariate analyses (“survival” R package). The LUAD patients in the validation set were also classified into high-/low-risk groups depending on the median CRG-risk score, followed by PCA and the generation of risk distribution diagrams and ROC curves. Uni- and multivariate analyses assessed the independent prognostic performance of the CRG-risk score in the validation set.

Lastly, the "survival, regplot, rms" R package was used to develop a nomogram of the factors aiding clinical prediction with prognostic values based on the Cox regression results. The accuracy of the nomogram in practical applications was assessed with a calibration chart.

### Enrichment and immunity analysis of the two risk score groups

GSEA (version 4.2.2) identified the significant gene set enrichment or depletion between the two risk groups. A "FDR < 0.05" cutoff value was utilized to identify the differential KEGG pathways. Additionally, the immune cell infiltration and immune functions of the two risk groups were analyzed employing the R software "limma, reshape2, ggpubr" package.

## Results

### Expression profile and mutation of CRGs in LUAD

**Fig. 2:**
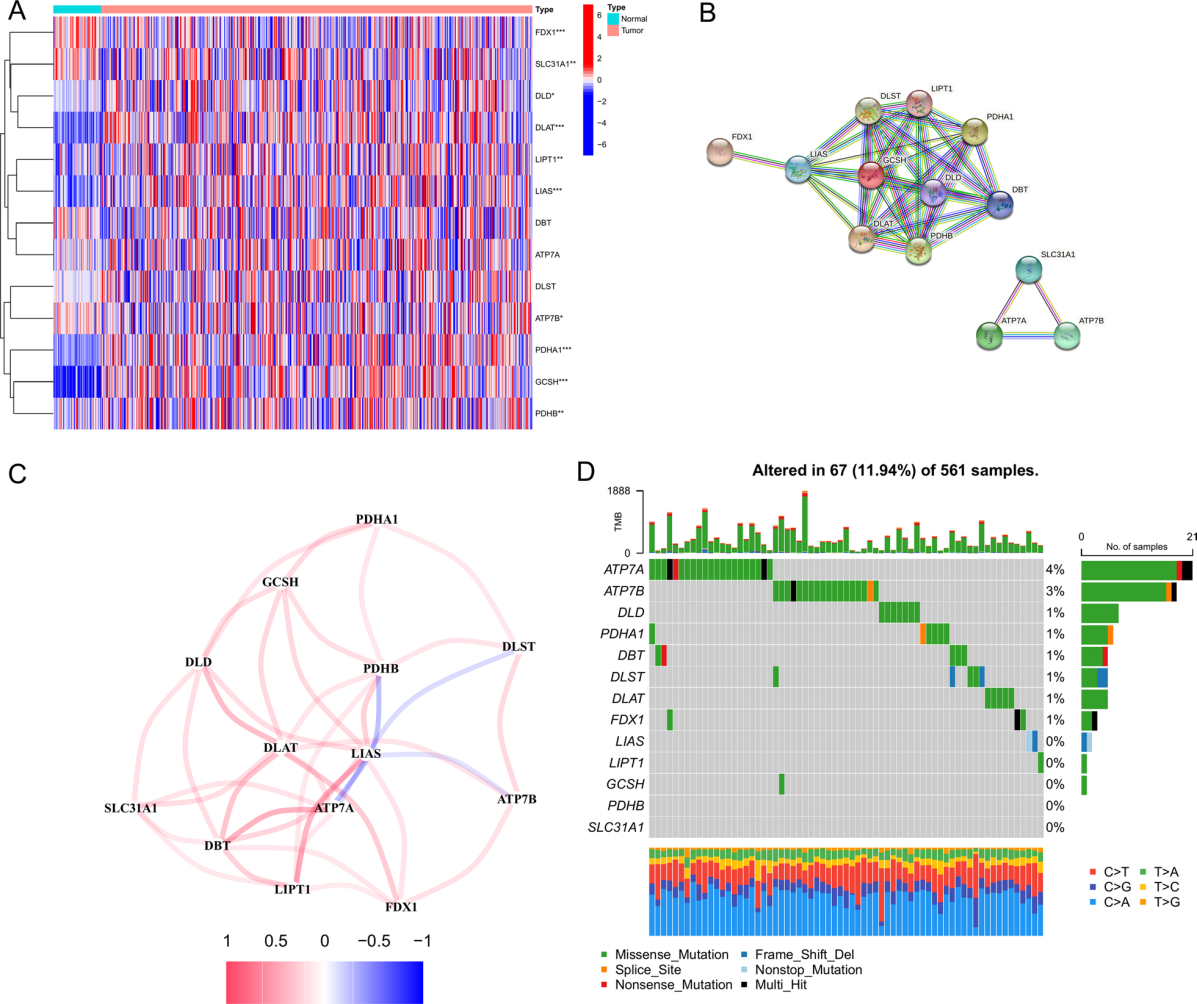
Expression patterns and mutation status of the CRGs. **A** Heatmap depicting the expression of CRGs in LUAD and normal tissues in the TCGA cohort. **B** The protein–protein interaction network of CRGs. **C** The co-expression network of CRGs. **D** A waterfall plot was showing the mutation status of CRGs in LUAD

In this study, the expression pattern of 13 CRGs was studied in LUAD and normal tissues, as visualized in a heat map (Fig. 2A). Protein–protein interaction network and correlation analyses of these CRGs (Figs. 2B, C) suggested inverse correlations of LIAS with PDHB, DLST, ATP7A, and ATP7B and positive correlations among the remaining CRGs. Mutation analysis of 13 CRGs revealed that ATP7A and ATP7B harbored the highest mutation frequency in LUAD, followed by DLD, PDHA1, DBT, DLST, DLAT, and FDX1, with missense mutation being the primary type of mutation. No significant mutation was observed in other CRGs (Fig. 2D).

### Identification of cuprotosis subtypes in LUAD

**Fig. 3:**
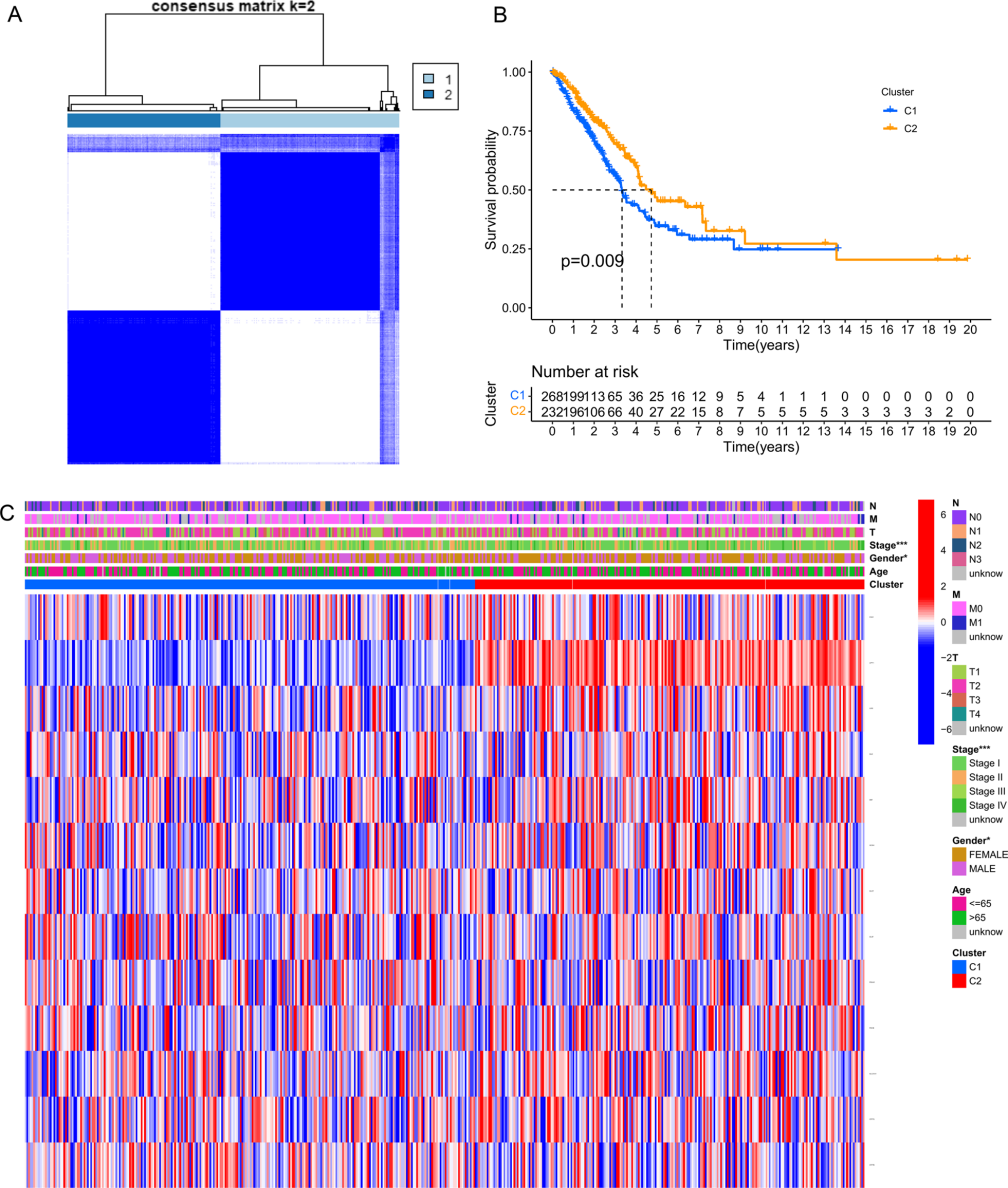
The molecular subtypes of CRGs based on clinicopathological features and prognosis. **A** Consensus Matrix Heatmap showing two molecular subtypes (k = 2). **B** The relation of CRGs molecular subtypes to the overall survival of LUAD patients. **C** The clinicopathological features and differential expression of CRGs in the two molecular subtypes

Next, we performed subtype classification of the TCGA-LUAD patients using a consensus clustering algorithm to identify the characteristics of the CRGs based on their expression profiles. This analysis divided TCGA-LUAD cohort into two molecular subtypes as the best choice (k = 2, Fig. 3A). A curve helped find the difference in survival between the two molecular subtypes, suggesting significantly longer OS of LUAD patients with molecular subtype C1 than those with molecular subtype C2 (p = 0.009; Fig. 3B). Significant differences were detected in the expression of CRGs and stage classification (***p < 0.001; Fig. 3C) upon comparing the clinicopathological prognostic parameters of the patients from two different molecular subtypes.

### DEGs between molecular subtypes of CRGs

**Fig. 4:**
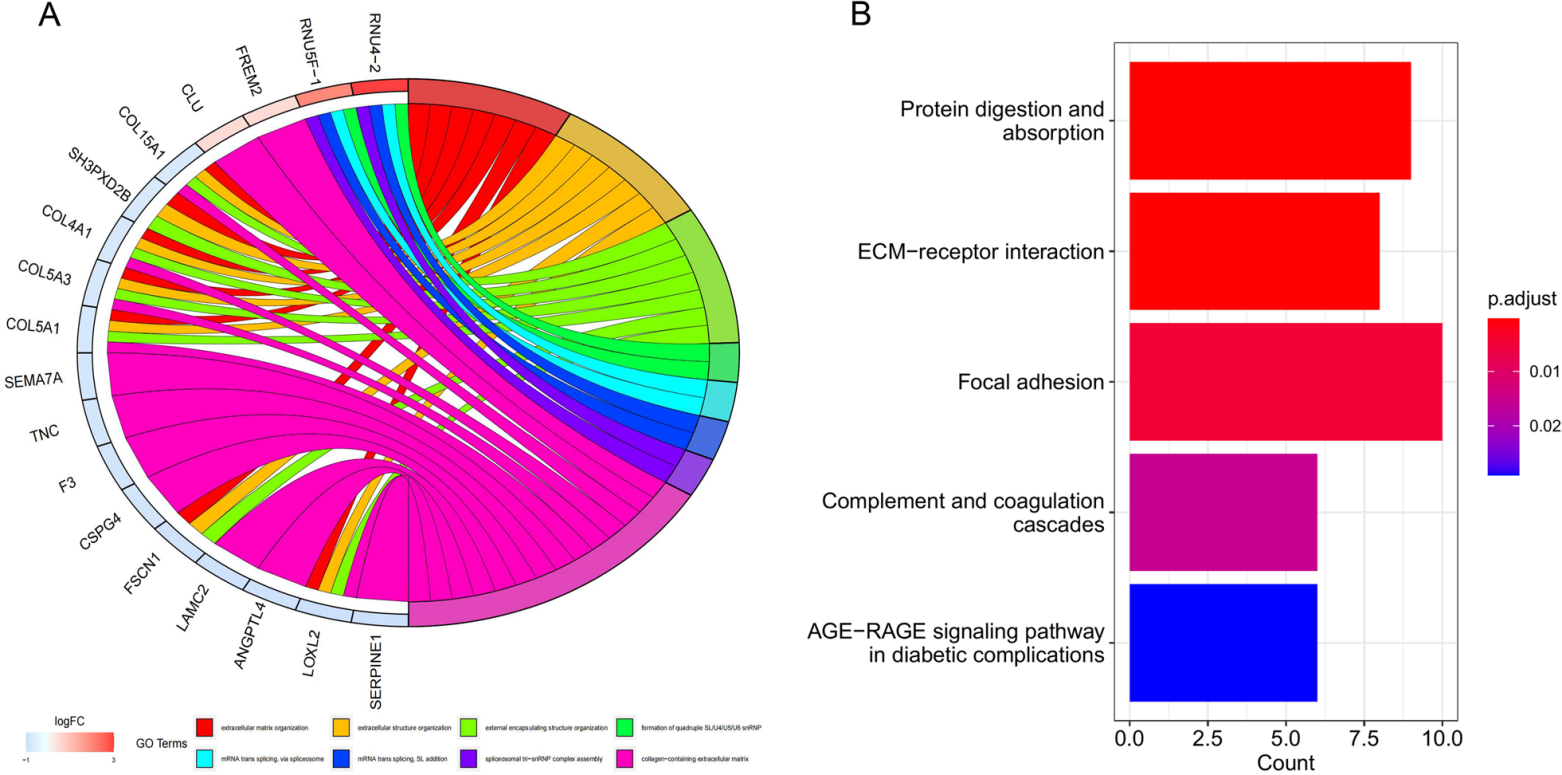
Enrichment analyses of DEGs between different molecular subtypes of CRGs. **A** Biological functional enrichment analysis. **B** Signaling pathway enrichment analysis

Differential expression analysis between the two molecular subtypes, based on the CRGs, identified 342 DEGs associated with cuprotosis subtypes. Functional and pathway enrichment analyses of the above-identified genes (Figs. 4A, B) revealed that these cuprotosis subtype-related DEGs were principally linked to functions such as the regulation of the extracellular matrix, mRNA trans-splicing, etc. Pathway enrichment analysis showed an association of the cuproptosis subtype-related DEGs with focal adhesion, ECM-receptor interaction, and protein digestion and absorption pathways[17].

### Construction and validation of a cuprotosis pattern-related prognostic model

In this study, we used TCGA-LUAD cohort as the training set and the GEO-GSE68465 cohort as the validation set. LASSO regression analysis was performed for 43 cuprotosis pattern-relevant DEGs with prognostic values, identified using univariate analysis of the training set. Finally, a prognostic model was developed using ten genes by maximally reducing the risk of overfitting. Risk scores were calculated for a prognostic model relating to cuprotosis patterns:

**CRGs-risk score** = (0.1231*GJB3_expression) + (0.0268*KIAA0319_expression) + (-0.0606*ZNF493_expression) + (0.0125*KCNF1_expression) + (-0.0869*MS4A1_expression) + (0.1068*FLNC_expression) + (0.0202*FSCN1_expression) + (0.0550*ANGPTL4_expression) + (0.0234*AHNAK2_expression) + (-0.0025*ELF5_expression).

**Fig. 5:**
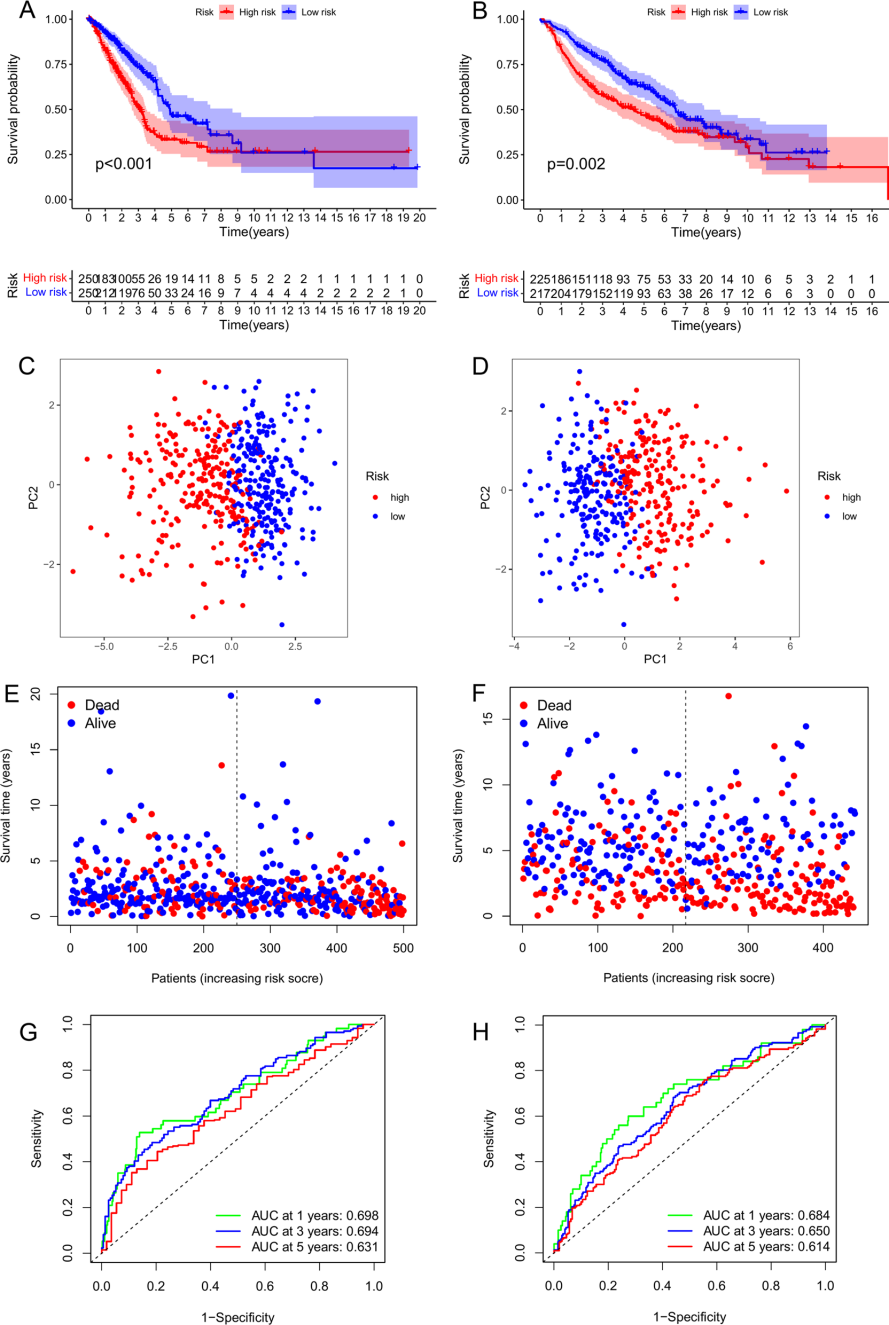
Internal and external validation of risk models. **A** Overall survival analysis of the high-/low-risk subgroups in the training set (TCGA cohort). **B** Overall survival analysis of the high-/low-risk subgroups in the validation set (GEO cohort). **C** Principal component analysis of the training set (TCGA cohort)t. (D) Principal component analysis of the validation set (GEO cohort). **E** Risk distribution map of the training sett (TCGA cohort). **F** Risk distribution map of the validation set (GEO cohort). **G** ROC curves of predictive performance in the training sett (TCGA cohort). (H) ROC curves of predictive performance in the validation set (GEO cohort)

**Fig. 6:**
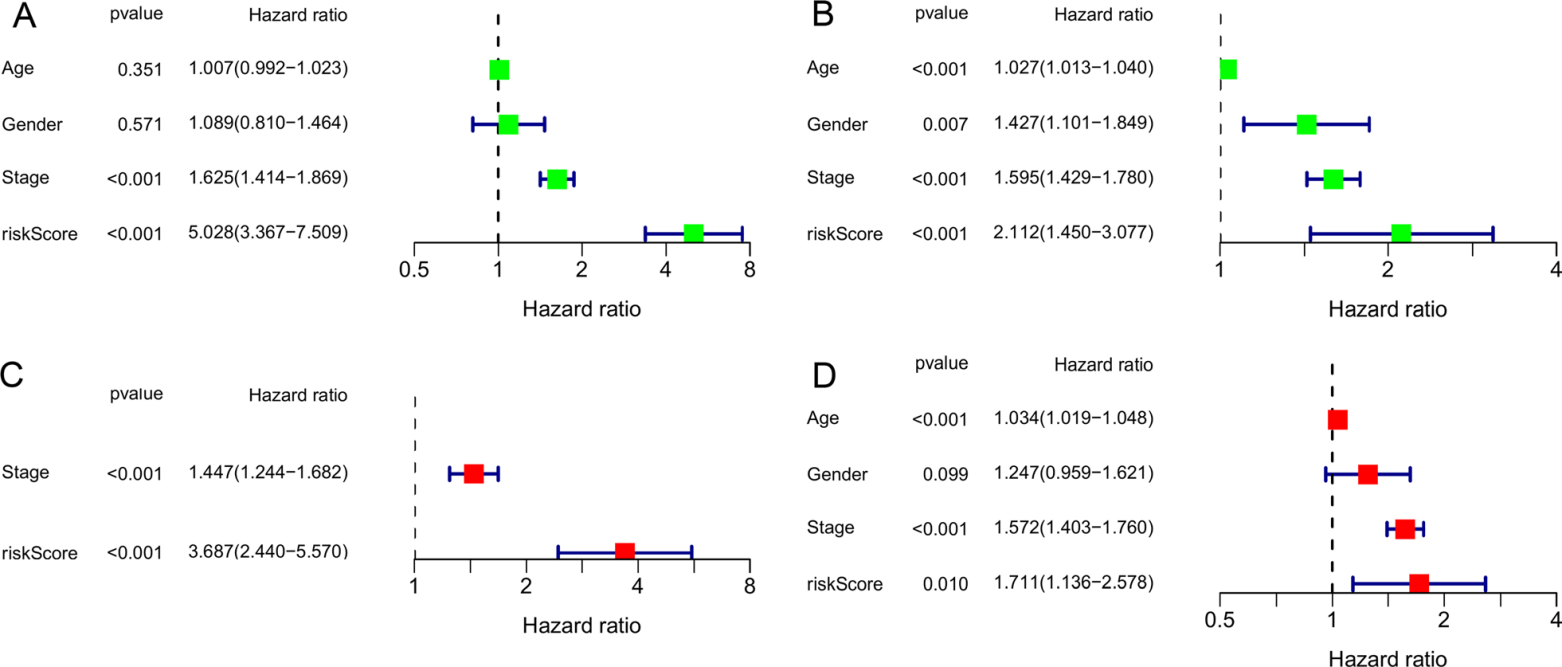
Independent prognostic value analysis of CRG-risk score. **A** Univariate analysis of the CRG-risk score in the training set. **B** Univariate analysis of the CRG-risk score in the validation set. **C** Multivariate analysis of the CRG-risk score in the training set. **D** Multivariate analysis of the CRG-risk score in the validation set

The LUAD patients in the training set were sub-divided into high-/low-risk groups with the median CRG-risk score as the threshold. The survival curves exhibited significant survival differences between the two groups (*p* < 0.001), with shortened survival time of the high-risk patients (Fig. 5A). The PCA results showed that the CRGs-risk score accurately distinguished high-/low-risk populations (Fig. 5C). The CRG-risk score-based risk distribution map showed that the mortality of patients increased with this score, while survival was prolonged in patients with lower scores (Fig. 5E). The area under the ROC curve (AUC) values of CRG-risk score predicting the 1-, 3-, and 5-year survival rates of LUAD patients were 0.698, 0.694, and 0.631, respectively, indicating a robust prediction of the LUAD patients’ survival based on the risk score of the model (Fig. 5G). We carried out uni- and multivariate analyses to determine whether the CRGs-risk score could serve as an independent prognostic indicator. The analyses revealed that the CRG-risk score performed well as an independent prognostic factor for LUAD (univariate: *p* < 0.001, HR [95% CI] = 5.028 [3.367, 7.509]; multivariate: *p* < 0.001, HR [95% CI] = 3.687 [2.440, 5.570]; Fig. 6A, C).

We used the external validation set (GEO-GSE68465 cohort) for validation to verify the predictive ability of the CRG-risk score. The risk score of the validation set was calculated using the same formula as the training set, which sub-classified the LUAD patients of the validation set into high-/low-risk groups based on the median value of the CRG-risk score. Analysis of the validation set revealed worse prognoses for the high-risk patients than the lower-risk individuals (*p* = 0.002; Fig. 5B). PCA showed the accuracy of the CRG-risk score in distinguishing the high-/low-risk groups (Fig. 5D). The risk distribution map indicated increased mortality and shortened survival of patients with high CRG-risk scores (Fig. 5F). The AUC curves for 1-, 3-, and 5-year survival rates were 0.684, 0.650, and 0.614, respectively, indicating an excellent predictive performance of the CRG-risk score (Fig. 5H). Subsequent uni- and multivariate analyses of the training set provided consistent findings indicating that the CRGs-risk score could be utilized as an independent prognostic indicator for LUAD (univariate: *p* < 0.001, HR[95%CI] = 2.112 [1.450, 3.077]; multivariate: *p* < 0.001, HR[95] %CI] = 1.711 [1.136, 2.578]; Fig. 6B, D).

### Construction and validation of the nomogram

**Fig. 7:**
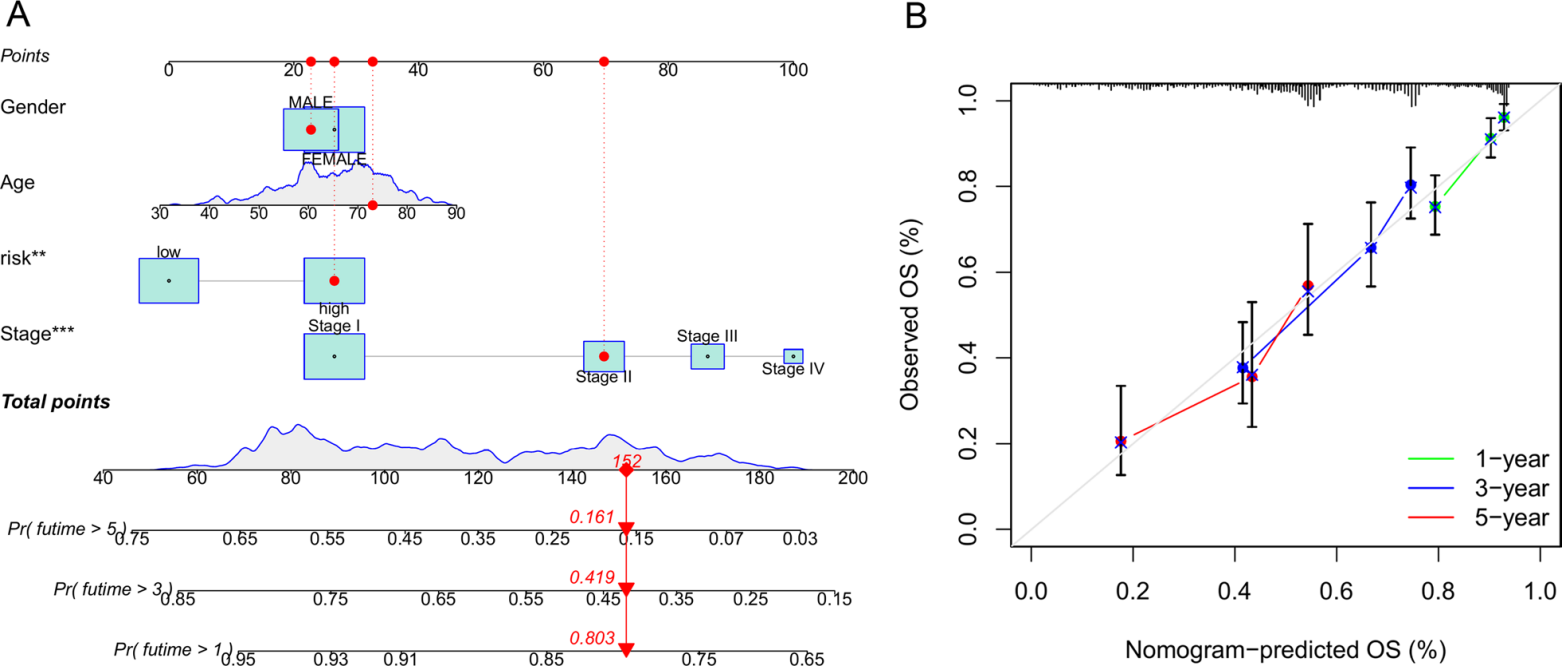
Nomogram establishment and validation. **A** Nomograms for the prognostic prediction of LUAD patients. **B** 1-year, 3-year, and 5-year calibration charts

A visual nomogram was developed as a multi-factor integrated tool based on CRG-risk score and clinical characteristics of patients, such as gender, age, and staging. The nomogram was applied for the prognostic prediction of LUAD patients (Fig. 7A). We next examined the clinical predictive performance of the nomograms using calibration plots. We found that the nomograms were robust and valid for clinically predicting 1-, 3-, and 5-year overall survival of LUAD patients, with the predicted results resembling actual clinical results (Fig. 7B).

### Enrichment analysis and immune activity assessment of the two risk score groups

**Fig. 8:**
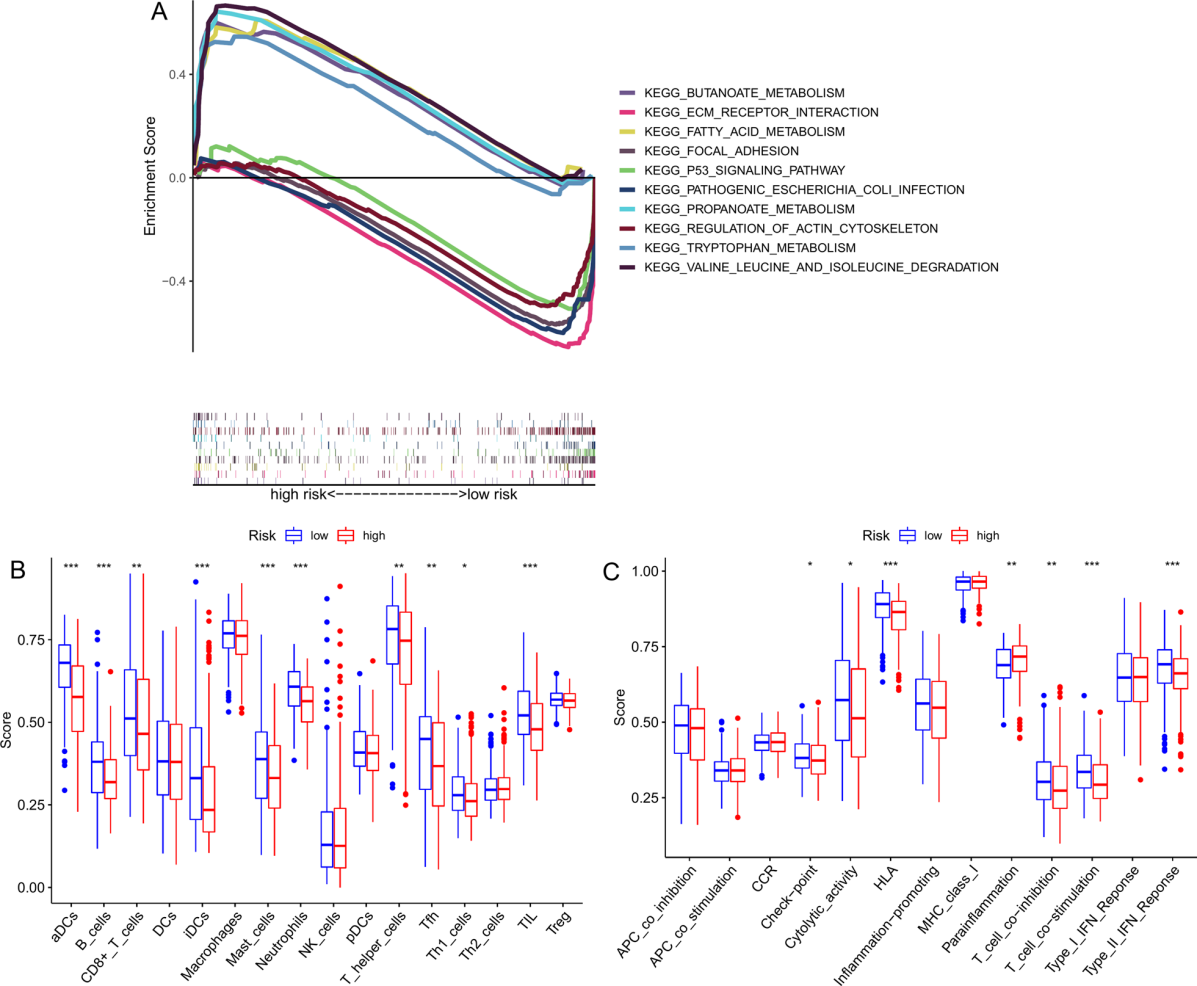
Enrichment analysis and immune activity of the two risk score subgroups. **A** Gene set enrichment analysis (GSEA). **B**, **C** Immune activity evaluation by single-sample GSEA (ssGSEA)

We performed GSEA analysis on the high-/low-risk patients grouped by the median CRG-risk score and selected five critical gene sets in each group with "*p-val* < 0.01" as a threshold. The enriched pathways in the high-risk group included focal adhesion, ECM-receptor interaction, p53 signaling pathway, regulation of actin cytoskeleton, and pathogenic *Escherichia coli* infection pathways; the low-risk-related pathways included degradation of valine, leucine, and isoleucine, fatty acid metabolism, propanoate metabolism, butanoate metabolism, and tryptophan metabolism pathways (Fig. 8A). Additionally, we used ssGSEA to evaluate the differences in immune cell infiltration and immune pathways between the high- and low-risk CRG-risk score groups. In comparison with the high-risk patients, increased infiltration of multiple immune cells (aDCs, B_cells, CD8 + _T_cells, etc.) was observed in the low-risk individuals (*p* < 0.05; Fig. 8B). Also, multiple immune functions such as HLA, T_cell_co-stimulation, Type_II_IFN_Response, etc., showed an immunosuppressive phenotype in the high-risk group (*p* < 0.05; Fig. 8C).

## Discussion

The increasing yearly incidence of lung cancers warrants early screening and comprehensive risk assessment. Despite many studies on the prognosis of lung cancer, the diagnosis and prognosis of lung cancer patients largely depend on the clinicopathological and TNM staging system (IASLC/AJCC eighth edition) [18, 19]. However, this staging system lacks sufficient sensitivity and individualization for the diagnosis and prognosis of lung cancer patients. Therefore, there is crucial to develop more effective and individualized diagnostic and prognostic evaluation tools [20]. At present, the pathogenesis of lung cancer is still being investigated and has not been entirely ascertained. Successfully catering to the challenges in treatment and prognosis requires an in-depth exploration of the molecular mechanism and genome of lung cancer to improve patient survival. Therefore, constructing a novel and effective gene prognostic model is critical to enhancing the efficacy of medication for improving the prognosis of lung cancer patients. Recently, some progress has been made in copper-related research, and the role of copper in tumors has been partly elucidated [21–23]. Researchers also used bioinformatic analysis to preliminarily examine the relationship between cuproptosis, tumor typing, and tumor microenvironment. Hence, this study comprehensively evaluated the expression profile of CRGs, identified novel CRG-based LUAD subtypes, and constructed a prognostic model for LUAD patients.

In the present study, we compared the expression of CRGs in LUAD patients and eight up-regulated and two down-regulated genes, of which three had no significant difference. Also, we unraveled the genetic changes in CRGs in LUAD and the correlations between these genes. We accurately and easily differentiated LUAD patients into two molecular subgroups based on the expression of the 13 CRGs, with significant differences in survival between the two molecular subtypes. Relative to C2 molecular subtype patients, the C1 patients showed better overall survival. Afterward, 342 DEGs were identified between the two cuproptosis molecular subtypes, which mainly regulated tumor invasion. We finally selected ten cuproptosis pattern-related DEGs with significant prognostic values to develop a prognostic model. As proven by the external validation set, the CRG-risk score of the prognostic model is robust and effective, with excellent predictive accuracy. Uni- and multivariate analyses revealed CRG-risk score as an independent prognostic marker for LUAD patients. Lastly, the CRG-risk score was integrated with multiple variables, such as clinicopathological characteristics, to create a quantitative nomogram for predicting the survival and prognosis of LUAD patients. Such a nomogram contributed to the better clinical application value of the CRG-risk score. The prognostic model constructed in this study is crucial for the stratification of LUAD patients, which is partly conducive to understanding the molecular mechanism underlying LUAD, directing improved targeted therapies against LUAD.

Furthermore, we unveiled that the high CRG-risk score group showed strong enrichment of gene sets like focal adhesion, ECM-receptor interaction, and P53 signaling pathway. Wu Y et al. have illustrated that enhancing EMT and focal adhesion leads to the development of LUAD [24]. Liu W et al. have also indicated that CX3CL1 induces the migration and invasion of lung cancer cells via the Src/focal adhesion signaling pathway [25]. Cell–matrix adhesion is crucial to multiple critical biological processes such as cell motility, proliferation, differentiation, regulation of gene expression, cell survival, etc. Conclusively, regulating focal adhesion might potentially boost lung cancer cell migration and invasiveness [26]. Like focal adhesion, ECM-receptor interactions also affect the proliferative, migratory, and invasive properties of lung cancer cells [27]. Activation of p53 is elicited by multiple stress signals involving DNA damage, oxidative stress, and activated oncogenes and is associated with cell cycle arrest, cell senescence, or apoptosis. Regulation of the p53 signaling pathway might trigger lung cancer cell apoptosis and cell cycle arrest [28, 29]. Hence, targeting such pathway genes in patients with high CRG-risk scores might suppress tumor cell proliferation, migration, and invasion, highlighting a potential direction for targeted therapies against LUAD. The low CRG-risk score group correlated with a series of metabolic pathways, the most significant of which included valine, leucine, and isoleucine degradation, fatty acid metabolism, and propanoate metabolism pathways. This observation indicated that regulating basal metabolism and ameliorating metabolic disorders will be conducive to anti-tumor activity [30, 31]. Meanwhile, the infiltration of immune cells (B cells, mast cells, etc.) in LUAD patients decreased, and immune function was suppressed in the high CRG-risk score group. The anti-tumor immune response mediated by immune cells is essential for tumor prevention [32]. Therefore, therapies that promote anti-tumor immune responses are considered helpful and effective in treating patients with LUAD.

However, some limitations also exist in this study: 1) the data used in the present study are all sourced from public databases (TCGA and GEO); 2) the model in this study is only validated by employing an external validation set, warranting additional experiments for validation.

## Conclusion

Our study bioinformatically explored the prognostic effect of CRGs in LUAD. Identifying new molecular subtypes of LUAD based on optimal cluster numbers of CRGs has helped generate new insights. We also successfully developed a prognostic model based on the CRGs-risk score relating to cuproptosis patterns. Integrating the CRGs-risk score with multiple variables, including clinicopathological characteristics, the quantitative nomogram is constructed, aiding the prognostic evaluation of LUAD patients in clinical diagnosis and treatment. In addition, the therapeutic effect of CRG-risk score in LUAD immunotherapy was determined. These findings will be conducive to guiding better targeting and immunotherapy in LUAD patients.

## Data Availability

The datasets used and analyzed during the current study are available from The Cancer Genome Atlas (TCGA, https://portal.gdc.cancer.gov/, TCGA-LUAD), and Gene expression Omnibus (GEO, https://www.ncbi.nlm.nih.gov/geo/, the accession codes: GSE68465).

## References

[1] Wen T, Song L, Hua S (2021). Perspectives and controversies regarding the use of natural products for the treatment of lung cancer. Cancer Med.

[2] Nasim F, Sabath BF, Eapen GA (2019). Lung cancer. Med Clin North Am.

[3] Bade BC, Dela Cruz CS (2020). Lung cancer 2020: epidemiology, etiology, and prevention. Clin Chest Med.

[4] Pallis AG, Syrigos KN (2013). Lung cancer in never smokers: disease characteristics and risk factors. Crit Rev Oncol Hematol.

[5] Bossé Y, Amos CI (2018). A decade of gwas results in lung cancer. Cancer Epidemiol Biomarkers Prev.

[6] Mao Y, Yang D, He J (2016). Epidemiology of lung cancer. Surg Oncol Clin N Am.

[7] Sung H, Ferlay J, Siegel RL (2021). Global cancer statistics 2020: GLOBOCAN estimates of incidence and mortality worldwide for 36 cancers in 185 countries. CA Cancer J Clin.

[8] Nooreldeen R, Bach H (2021). Current and future development in lung cancer diagnosis. Int J Mol Sci.

[9] de Sousa VML, Carvalho L (2018). Heterogeneity in lung cancer. Pathobiology.

[10] Parikh AR (2019). Lung cancer genomics. Acta Med Acad.

[11] D'Arcy MS (2019). Cell death: a review of the major forms of apoptosis, necrosis and autophagy. Cell Biol Int.

[12] Tsvetkov P, Coy S, Petrova B (2022). Copper induces cell death by targeting lipoylated TCA cycle proteins. Science.

[13] Rieber M (2020). Cancer Pro-oxidant therapy through copper redox cycling: repurposing disulfiram and tetrathiomolybdate. Curr Pharm Des.

[14] Denoyer D, Clatworthy SAS, Cater MA. Copper complexes in cancer therapy. Met Ions Life Sci. 2018;18:/books/9783110470734/9783110470734–022/9783110470734–022.xml. 10.1515/9783110470734-022 29394035

[15] Kadu P, Sawant B, Kale PP, Prabhavalkar K (2021). Copper-lowering agents as an adjuvant in chemotherapy. Indian J Pharmacol.

[16] Liu T, Cai L, Hua H (2022). Cuprotosis patterns are associated with tumor mutation burden and immune landscape in lung adenocarcinoma. J Oncol.

[17] Kanehisa M, Furumichi M, Tanabe M, Sato Y, Morishima K (2017). KEGG: new perspectives on genomes, pathways, diseases and drugs. Nucleic Acids Res.

[18] Feng SH, Yang ST (2019). The new 8th TNM staging system of lung cancer and its potential imaging interpretation pitfalls and limitations with CT image demonstrations. Diagn Interv Radiol.

[19] Carter BW, Lichtenberger JP, Benveniste MK (2018). Revisions to the TNM staging of lung cancer: rationale, significance, and clinical application. Radiographics.

[20] Herbst RS, Morgensztern D, Boshoff C (2018). The biology and management of non-small cell lung cancer. Nature.

[21] Li H, Wang J, Wu C (2020). The combination of disulfiram and copper for cancer treatment. Drug Discov Today.

[22] da Silva DA, De Luca A, Squitti R (2022). Copper in tumors and the use of copper-based compounds in cancer treatment. J Inorg Biochem.

[23] Wang W, Wang X, Luo J (2021). Serum copper level and the copper-to-zinc ratio could be useful in the prediction of lung cancer and its prognosis: a case-control study in northeast china. Nutr Cancer.

[24] Wu Y, Liu L, Shen X (2021). Plakophilin-2 promotes lung adenocarcinoma development via enhancing focal adhesion and epithelial-mesenchymal transition. Cancer Manag Res.

[25] Liu W, Liang Y, Chan Q (2019). CX3CL1 promotes lung cancer cell migration and invasion via the Src/focal adhesion kinase signaling pathway. Oncol Rep.

[26] Koetsier JL, Amargo EV, Todorović V (2014). Plakophilin 2 affects cell migration by modulating focal adhesion dynamics and integrin protein expression. J Invest Dermatol.

[27] Que ZJ, Yang Y, Liu HT (2021). Jinfukang regulates integrin/Src pathway and anoikis mediating circulating lung cancer cells migration. J Ethnopharmacol.

[28] Zhao Y, Cai J, Shi K (2021). Germacrone induces lung cancer cell apoptosis and cell cycle arrest via the Akt/MDM2/p53 signaling pathway. Mol Med Rep.

[29] Ouyang L, Yang M, Wang X (2021). Long non-coding RNA FER1L4 inhibits cell proliferation and promotes cell apoptosis via the PTEN/AKT/p53 signaling pathway in lung cancer. Oncol Rep.

[30] Lu H, Li Y, Zhang H (2021). Direct quantitative profiling of amino acids in tissues for the assessment of lung cancer. Talanta.

[31] Chen W, Li Q, Hou R, et al. An integrated metabonomics study to reveal the inhibitory effect and metabolism regulation of taurine on breast cancer [published online ahead of print, 2022 Mar 6]. J Pharm Biomed Anal. 2022;214:114711. 10.1016/j.jpba.2022.114711 35306435

[32] Raposo TP, Beirão BC, Pang LY (2015). Inflammation and cancer: till death tears them apart. Vet J.

